# Determining Seed Viability During Fruit Maturation to Improve Seed Production and Availability of New Citrus Rootstocks

**DOI:** 10.3389/fpls.2021.777078

**Published:** 2021-11-19

**Authors:** Deived Uilian de Carvalho, Daniel A. Boakye, Tim Gast, Rui Pereira Leite Junior, Fernando Alferez

**Affiliations:** ^1^Southwest Florida Research and Education Center, Institute of Food and Agricultural Sciences, University of Florida, Immokalee, FL, United States; ^2^Department of Crop Protection, Instituto de Desenvolvimento Rural do Paraná, Londrina, Brazil

**Keywords:** growth rate, nursery assessment, rootstocks, seed germination, seed vigor

## Abstract

In recent years, the pressure for replanting and resetting huanglongbing (HLB or citrus greening) affected citrus groves has led to an inadequate seed supply for the most popular rootstock cultivars in the State of Florida, United States. Early fruit harvesting of citrus rootstock source trees might reduce fruit losses and enhance seed availability, especially in HLB-endemic and hurricane susceptible areas, if the physiological quality of the seeds is adequate. The effects of fruit maturity on seed quality and seedling performance of US-802, US-897, and US-942 citrus rootstocks were investigated for two consecutive growing seasons. The study included the evaluation of seed germination and nursery performance of the citrus rootstock seedlings. The germination test was performed *in vitro*, where seeds were hand-peeled, surface-sterilized and placed in culture tubes containing basal Murashige and Skoog medium. For the emergence test, seeds were sown in seedling trays containing sterilized growing substrate in a greenhouse with controlled-environment conditions. Rootstock fruits from all three varieties harvested in August and September had seeds with higher germination potential, as more than 90% of the seeds generated seedlings. US-942 had more % of emergence than US-802 and US-897, resulting in faster seed germination; in contrast, US-802 had the faster shoot growth rate. Assays on fruit abscission response showed that by August, fruit from all three varieties were responsive to ethylene and abscised, although response varied and was higher in US-942, suggesting the seeds were mature enough. Taken together, our findings indicate that fruits these three rootstocks can be harvested as early as August in contrast to the current procedures without losing germination potential. This will result in an increase in available seeds for nurseries in Florida.

## Introduction

Currently, Florida’s citriculture faces an unprecedented crisis due to the widespread presence of citrus greening or Huanglongbing (HLB), a disease associated with the phloem-limited bacterium *Candidatus* Liberibacter asiaticus that has become endemic in the State. Citrus trees affected by HLB are characterized by root loss, branch die-back, yellow shoots, blotchy mottle chlorotic patterns on leaves, and off-tasting and malformed fruit. HLB infection inhibits root growth, reduces nutrient uptake, and promotes leaf and fruit drop, and whole tree decline that is often lethal (Pustika et al., 2008; Etxeberria et al., 2009). HLB also plays a major role in several aspects of citrus production, including the choice of the citrus rootstock. Replanting HLB-affected trees with disease-free nursery trees may require rootstocks resistant to soil diseases as *Phytophthora* gummosis, in order to guarantee the reestablishment of the grove (Graham et al., 2013).

As mature trees decline due to HLB, there is an increasing demand of citrus rootstocks for replanting. Under this pressure for replanting and resetting HLB-affected groves, there is a clear concern among citrus nursery operators and growers on seed availability of highly demanded rootstocks in Florida. Currently, seed propagation remains the preferred method of rootstock production in commercial citrus nurseries (Albrecht et al., 2020; Pokhrel et al., 2021), in part because there is no evidence on the vertical transmission of viable cells of the HLB pathogen “*Candidatus* Liberibacter spp.” through seeds to citrus seedlings (Belasque Júnior et al., 2009; Bagio et al., 2020), and also because the extended belief that different root architectures arising from vegetative propagation will result in trees of inferior quality as compared to those from seeds (Albrecht et al., 2020); as a result, over 80% of the licensed citrus nurseries prefer uniform liners from seeds rather than from tissue culture sources (Chaires, 2017). In any case, vigorous seeds are necessary to produce strong rootstocks. Seed vigor depends on seed maturation and can also be influenced by the extent of cold storage (Carvalho and Silva, 2013). In general, the more mature a seed, the longer it can be stored.

Since rootstock fruits are not edible and/or commercially exploited, little to no attention has been paid to fruit development in rootstock seed cultivars, and as a result, the minimum fruit maturation stage at which citrus rootstock seeds can germinate is unknown. In addition, natural calamities such as tropical storms and hurricanes may affect the number of seeds annually available in Florida (Albrigo et al., 2005; Alferez and Bordas, 2019). The Atlantic hurricane season peaks around September 10th, whereas historically, rootstock fruit harvesting for seed extraction has been performed between October and December. This has resulted in fruit loss and reduction in seed yield due to fruit drop caused by high-speed winds. For instance, Hurricane Irma on September 10th, 2017, severely affected the ability of the industry in Florida to produce adequate number of seeds necessary to satisfy nurseries needs that year. In the hardest hit areas, there was massive fruit drop, especially from the US-802, US-812, US-897, and US-942 rootstock varieties (Alferez, 2018). Seed availability from the SWFREC Immokalee, USDA Fort Pierce, and Whitmore Foundation in Leesburg, the main certified seed suppliers in Florida, decreased by 66% as compared to the previous season, resulting in a shortage in seed availability. This subsequently affected the whole supply chain leading to a reduction in replanting. In this scenario, it will be advantageous to know in advance when the fruit contains viable seeds. This would allow making informed decisions on when to harvest the fruit of each variety, facilitating to work around the peak of the hurricane season.

The information currently available on the effect of fruit age on physiological responses of seedlings that germinated from seed obtained from fruit at different time of the season is very limited (Orbovic et al., 2011, 2013). Since vigorous seed germination and establishment of seedlings with well-defined root growth are necessary for the development of a healthy nursery stock, it is imperative that healthy disease-free seeds with good vigor and viability are utilized to establish a nursery population that can in turn result in vigorous scion growth and rapid establishment in the field. For this it is necessary to know when a seed is mature enough to germinate and with good storability potential. Citrus rootstock fruit, as any other citrus fruit, naturally develop their abscission potential as they mature, i.e., when seeds are able to germinate, as abscission is a natural mechanism for seed dispersion. If harvesting is delayed, this results in a decrease in yield, as the fruit naturally starts to drop.

For all the above reasons it would be advantageous for the citrus nursery industry to find markers of seed maturity that allow harvesting the fruit from these rootstocks when the seed is able to germinate. In this paper we report the evaluation of several candidate markers for seed viability, including fruit color, size, and abscission capacity. Then, we study seed vigor as related to maturation stage. We show that sensitivity to abscission induction is a good marker for seed viability, allowing advancing fruit harvesting significantly without loss of seed vigor, and resulting in better yield of seeds of highly demanded citrus rootstocks in Florida.

## Materials and Methods

### Plant Material

Citrus hybrid rootstock trees from three different varieties planted at the Southwest Florida Research and Education Center, Immokalee, FL, United States, were used in the study as source of fruits and seeds. Fruit samples were collected from eight to 10-year-old grafted seed trees of US-802 (27 trees), US-897 (26 trees), and US-942 (27 trees) for the 2018 (mid-May, June, July, August, and September), 2019 (mid-December), and 2020 (mid-July, August, and September) cropping seasons and immediately transported to the laboratory for further analysis. In 2018, four replicates of 10-fruit sample were randomly selected at 1–2 m height from all rootstock variety trees at each evaluated period for the *in vitro* fruit abscission assay. Later, a batch of 200-fruit were randomly selected at 1–2 m height from all evaluated rootstock variety trees and divided into four replicates of 50 fruits at each harvest date for the 2019 and 2020 cropping seasons, to characterize the rootstock fruits and seeds and to evaluate the seed germination and seedling emergence performance. US-802 rootstock is the result of a cross of “Siamese” pummelo [*Citrus maxima* (Burn.) Merr.] × “Gotha Road” trifoliate orange [*Poncirus trifoliata* (L.) Raf.] while “US-897” originated from a cross of “Cleopatra” mandarin [*Citrus reshni* (Hort.) ex Tan.] × “Flying Dragon” trifoliate orange [*P. trifoliata* (L.) Raf.], both were officially released in 2007 by the Agricultural Research Service (ARS) of the U.S. Department of Agriculture (USDA). Later, in 2010, the “US-942” rootstock was also released. This rootstock selection was originated from a cross of “Sunki” mandarin [*Citrus sunki* (Hort.) ex Tan.] × “Flying Dragon” trifoliate orange [*P. trifoliata* (L.) Raf.]. These three are among the most sought-after rootstocks in Florida due to their relative novelty and better performance under endemic HLB conditions.

### Fruit Characterization

Four replicates of 10 fruits per rootstock were characterized according to their physical parameters at each fruit harvest date. Fruit were weighed (g) using a semi-analytical scale (Radwag, PS 1000.R2, Radom, Poland) and the fruit length (mm) and diameter (mm) were assessed with a digital caliper (Fowler High Precision, Inc., 54-101-150-2, Newton, MA, United States). The fruit shape was determined based on the length/diameter ratio. The fruit color development was monitored during the evaluated period using a portable colorimeter (Konica Minolta, CR-400, Tokyo, Japan). For this, four replicates of five fruit per rootstock were measured and three different readings were obtained along the equatorial circumference of each fruit.

The CIE L*a*b* color scale was adopted (McGuire, 1992), and the citrus color index (CCI) was calculated according to Jiménez-Cuesta et al. (1981):


$$C\,C\,I=\frac{1000\times a^{*}}{L^{*}\times b^{*}}$$

where, *CCI* = citrus color index, a* = red-green color value, b* = yellow-blue color value, L* = lightness.

The CCI is a comprehensive indicator for color impression with positive values for red, negative values for blue-green, and 0 for an intermediate mixture of red, yellow, and blue-green (Zhou et al., 2010). Lightness (L*) value ranges from 0 to 100 in which higher values indicate lighter color intensity (McGuire, 1992).

### Seed Extraction and Characterization

Seeds were hand-extracted from each rootstock fruits, washed under distilled water, and air-dried at room-temperature for 24 h. The number of filled seeds per fruit was counted using four replicates of 10-fruit per rootstock, collapsed and aborted seeds were not included. Seed length and width were measured for each rootstock using a digital caliper (Fowler High Precision, Inc., 54-101-150-2, Newton, MA, United States), and the seed weight was assessed using an analytical scale (Radwag, AS 60/220.R2, Radom, Poland). All seed measurements were based on four replicates of 100-seed sample.

### *In vitro* Seed Germination Evaluation

Seeds from all three rootstocks were individually peeled, standardized for the moisture content for 24 h under controlled-environment conditions (10°C, 100% RH, and dark), surface-sterilized for 20 min in a solution with 5.0% (v/v) sodium hypochlorite (Clorox Co., Oakland, CA, United States) and 0.01% (v/v) Tween 20 (Sigma-Aldrich, St. Louis, MO, United States), rinsed three times in distilled sterile water, and then sown. The germination test was performed *in vitro* in a complete randomized design using four replicates of 15 peeled seeds extracted from a 50-fruit sample for each harvest date and rootstock, as previously described. One sterile seed was individually placed per culture tube (25 × 150 mm) filled with 18 mL of MS (Murashige and Skoog, 1962) basal medium supplemented with 3% sucrose (w/v) and 0.7% agar (w/v) (Murashige and Skoog Basal Medium, M9274, Sigma-Aldrich, St. Louis, MO, United States), previously adjusted to pH of 5.8 and autoclaved at 121°C for 15 min. After sown, the culture tubes were sealed and maintained in growth chamber (Conviron Ltd., CMP6010, Winnipeg, MB, Canada) at constant temperature of 25 ± 1°C and dark for 30 days. Measurements were taken daily after root protrusion in order to calculate the total percentage of germination, root growth rate (RGR), shoot growth rate (SGR), and germination speed index (GSI) (germination rate) that measures the speed of germination and quantifies the seed vigor based on a time-weighted cumulative germination (Brown and Mayer, 1986). The percentage of multiple seedlings per seed (polyembryony) was quantified at the end of the germination evaluation. To calculate the root and SGR we used the following formula:


$$G\,r\,o\,w\,t\,h\,r\,a\,t\,e=\frac{\left|a_{2}-a_{1}\right|+\left|a_{3}-a_{2}\right|+\cdots +\left|a_{n}-a_{n-1}\right|}{n-1}$$

where a_1_, a_2_,…, a_(*n*)_, a_(*n*–1)_ = root or shoot length at corresponding day; and n = number of days. Growth rate was expressed in mm day^–1^.

The GSI was calculated based on the mathematical expression proposed by Maguire (1962):


$$G\,S\,I=\frac{g_{1}}{n_{1}}+\frac{g_{2}}{n_{2}}+\cdots +\frac{g_{n}}{n_{n}}$$

where GSI = germination speed index; *g*_1_, *g*_2_,… *g*_*n*_ = number of germinated seed recorded at the first count, second count,… and last count; *n*_1_, *n*_2_,… *n*_*n*_ = number of days seed have been sown at the first, second,… and last count.

### Seedling Emergence Evaluation

Nursery performance assessment of citrus rootstock seedlings was also evaluated using standard nursery methods. The emergence study was set in a complete randomized design using four replicates of 48 seeds extracted from a 50-fruit sample for each harvest date and rootstock, as previously described. One seed per tray cell was planted at 0.5 cm depth into seedling trays of 16.8 × 35.6 × 61.0 cm with 96 cells (Stuewe and Sons, Inc., FT96-7, Tangent, OR, United States) containing sterilized growing medium composed by sphagnum peat moss (Premier Horticulture, Inc., Pro-Mix HP Mycorrhizae, Quakertown, PA, United States). The trays were set on benches inside a controlled-environment greenhouse at the Southwest Florida Research and Education Center (Immokalee, FL, United States) and irrigated daily. The number of emerging seedlings was monitored daily to determine the number of days required to emerge 50% of the population. After 60 days of planting, when seeds ceased emerging, the total percentage of emerged seedlings was determined.

### *In vitro* Fruit Abscission Assay

Additionally, four replicates of 10 fruits per rootstock and treatment, containing the stem (2 cm), were harvested at each harvest date and immediately transported to the lab. A disposable plastic 3 mL Pasteur pipette was attached to the stem and the union sealed with parafilm to prevent leakage. Treatment solutions were applied through the pipette by cutting the upper portion of the pipette. Treatments were water (control) and 1-aminocyclopropane-l-carboxylic acid (ACC; 0.1 mM), as previously described (Merelo et al., 2017). Abscission rate was monitored daily.

### Statistical Analysis

The experimental design was completely randomized with a factorial arrangement [three treatments (rootstocks) × four seed extraction periods] replicated four times. The fruit characterization was performed using 10-fruit sample per replicate while 100-seed sample was taken per replicate for seed characterization. The evaluation of seed germination was based on 15-seed sample per replicate and 48-seed sample for the seedling emergence test. These data were submitted to normality and homogeneity of variances tests before analysis of variance (ANOVA) using the ExpDes package (Husson et al., 2017) in *R* v. 4.0.2 (The *R* Foundation for Statistical Computing, Vienna, Austria). Means were compared by Tukey’s test and taken to be significantly different at *P* ≤ 0.05. The correlation matrices were created for each citrus rootstocks based on the studied traits using the FactoMineR (Lê et al., 2008) and corrplot (Wei et al., 2017) *R* packages. Fruit abscission data from *in vitro* assays was expressed as mean ± std and was subjected to analysis of variance for means separation.

## Results and Discussion

### Seed Production

**FIGURE 1:**
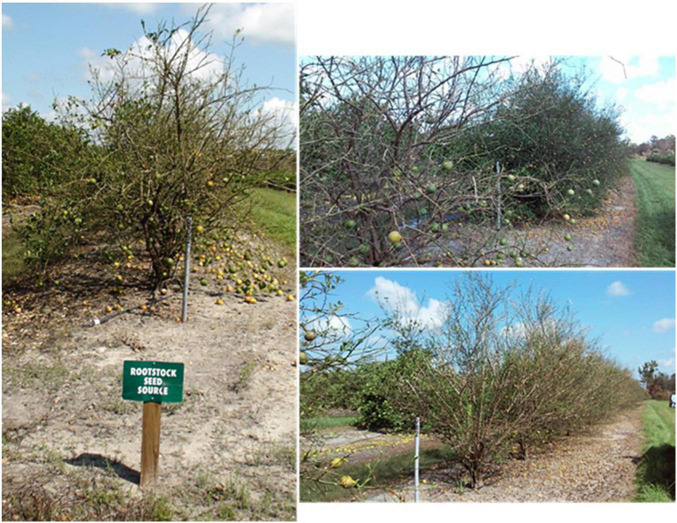
The rootstock seed source plantings at UF-IFAS Southwest Florida Research and Education Center in the aftermath of Hurricane Irma on September 14th, 2017. Note how virtually all rootstock seed-producing fruit from US-802, US-897, and US-942 was lost (Adapted from Alferez et al., 2021a).

On September 10th of 2017, hurricane Irma crossed the Florida Peninsula from South to North, affecting the seed production of major commercial citrus rootstocks due to tree severe damage and intense fruit drop (Figure 1). Seed availability from the three main registered rootstock seed sources in Florida, Southwest Florida Research and Education Center (Immokalee, FL, United States), U.S. Department of Agriculture (Ft. Pierce, FL, United States), and A.H. Whitmore Foundation Farm (Leesburg, FL, United States) decreased by 66% as compared to previous season, resulting in a shortage in seed availability (Table 1).

**TABLE 1:** Seed production of three commercial citrus rootstocks in Florida during two consecutive years, 2016 (no hurricane incidence) and 2017 (hurricane incidence).

Seed Source	Rootstock	2016			2017		
		Extraction	Boxes of fruits	Seed (liter)	Extraction	Boxes of fruits	Seed (liter)
SWFREC^1^ (Immokalee, FL, United States)	US-802	December	40	72	October	15	41
	US-897	December	38	84	October	16	60
	US-942	December	18	28	October	4	18
USDA HRL^2^ (Ft. Pierce, FL, United States)	US-802	December	13	23	November	6	10
	US-897	December	18	40	November	9	20
	US-942	December	10	16	November	4	10
A.H. Whitmore Foundation (Leesburg, FL, United States)	US-802	December	27	48	November	6	10
	US-897	December	23	51	November	6	14
	US-942	December	10	16	November	5	10
Total (All locations)	US-802	–	80	143	–	27	61
	US-897	–	79	175	–	31	94
	US-942	–	38	60	–	13	38

*^1^Southwest Florida Research and Education Center.*

*^2^U.S. Department of Agriculture, Horticultural Research Laboratory.*

### Fruit and Seed Characterization

Significant interaction between rootstock and time of harvest was observed for fruits and seeds (Tables 2, 3). In all three rootstocks studied growth pattern was similar, and typically sigmoidal, as described elsewhere for citrus (Agustí and Primo-Millo, 2020). In September 2020, fruits had the highest weight and size. These values were similar or even higher than those observed in December 2019 when the fruits were fully mature (Table 2). The characteristics of the fruits may vary with the cropping season, due to biotic and abiotic factors, such as HLB (Rosales and Burns, 2011) or water stress (Garcia-Tejero et al., 2011), and may also depend on the fruit loading (Iglesias et al., 2007; Agustí and Primo-Millo, 2020). Fruits from US-802 were larger than those from US-897 and US-942, and had the highest number of seeds per fruit (33–50), depending on the season (Table 3). Number of seeds was different depending on the rootstock variety and fruit size, as previously reported (Alferez and Bordas, 2019). The number of seeds per fruit is one of the major factors for citrus rootstock selection. Citrus rootstocks that produce large number of seeds per fruit and good seedling emergence performance are preferred by the nurseries (Bisi et al., 2020); however, it is worth noting that US-942, one of the rootstocks currently most favored by citrus growers in Florida due to its superior performance under HLB conditions, produces significantly less seeds (Table 3). This adds an additional challenge to the citrus industry.

**TABLE 2:** Fruit characterization of three citrus rootstocks harvested during different period at the Southwest Florida Research and Education Center, Immokalee, FL, United States (mean value ± standard deviation).

Source of variance	Fruit weight (g)			
Rootstock	July	August	September	December
US-802	178 ± 2.88 Da^1^	194 ± 4.78 Ca	249 ± 2.78 Aa	229 ± 3.81 Ba
US-897	29 ± 1.30 Cb	34 ± 2.40 Bb	41 ± 2.29 Ab	32 ± 2.34 BCc
US-942	25 ± 0.94 Db	31 ± 1.13 Cb	43 ± 2.83 Bb	50 ± 1.31 Ab
CV (%)	2.79			
Rootstocks	***			
Harvest	***			
Rootstock × Harvest	***			

	**Fruit length (mm)**			

US-802	69.4 ± 1.20 Ba	69.7 ± 1.41 Ba	75.4 ± 1.62 Aa	74.3 ± 2.15 Aa
US-897	33.2 ± 0.55 Bb	35.1 ± 0.65 Bb	37.9 ± 0.90 Ac	35.0 ± 0.81 Bc
US-942	33.7 ± 0.49 Bb	36.1 ± 0.57 Bb	40.6 ± 1.08 Ab	41.3 ± 2.14 Ab
CV (%)	2.62			
Rootstocks	***			
Harvest	***			
Rootstock × Harvest	***			

	**Fruit diameter (mm)**			

US-802	76.0 ± 0.67 Ba	76.7 ± 1.22 Ba	82.7 ± 1.08 Aa	78.1 ± 2.71 Ba
US-897	39.4 ± 0.75 Bb	41.4 ± 1.08 ABb	43.5 ± 0.24 Ab	40.7 ± 1.28 Bc
US-942	36.2 ± 0.55 Dc	39.2 ± 0.56 Cc	43.5 ± 0.98 ABb	45.8 ± 1.16 Ab
CV (%)	2.22			
Rootstocks	***			
Harvest	***			
Rootstock × Harvest	***			

	**Fruit shape (length/diameter)**			

US-802	0.91 ± 0.01 Ba	0.91 ± 0.01 Ba	0.91 ± 0.01 Ba	0.95 ± 0.01 Aa
US-897	0.84 ± 0.01 Ab	0.85 ± 0.01 Ab	0.87 ± 0.01 Ab	0.86 ± 0.01 Ac
US-942	0.93 ± 0.01 Aa	0.92 ± 0.01 ABa	0.93 ± 0.01 Aa	0.90 ± 0.03 Bb
CV (%)	1.64			
Rootstocks	***			
Harvest	ns			
Rootstock × Harvest	***			

*^1^Means followed by the same capital and lowercase letter in the row and column, respectively, do not significantly differ according to the Tukey’s test (*P* ≤ 0.05).*

Significant level: ***P ≤ 0.001; ns, non-significant.

**TABLE 3:** Characterization of three citrus rootstock seeds extracted during different period at the Southwest Florida Research and Education Center, Immokalee, FL, United States (mean value ± standard deviation).

Source of variance	Seeds per fruit			
Rootstock	July	August	September	December
US-802	46 ± 2.59 Ba	46 ± 1.12 Ba	50 ± 1.28 Aa	33 ± 1.53 Ca
US-897	19 ± 1.11 Ab	16 ± 2.23 Bb	19 ± 0.84 Ab	17 ± 1.37 ABb
US-942	15 ± 0.88 Ac	13 ± 1.76 ABc	13 ± 1.32 ABc	12 ± 1.05 Bc
CV (%)	6.07			
Rootstocks	***			
Harvest	***			
Rootstock × Harvest	***			

	**Seed length (mm)**			

US-802	13.9 ± 0.52 Ba	15.1 ± 0.29 Aa	14.5 ± 0.62 ABa	14.3 ± 0.53 Ba
US-897	8.7 ± 0.26 Bc	10.3 ± 0.16 Ac	10.2 ± 0.19 Ac	9.9 ± 0.18 Ac
US-942	10.5 ± 0.33 Bb	11.2 ± 0.31 ABb	11.3 ± 0.48 ABb	11.5 ± 0.20 Ab
CV (%)	3.19			
Rootstocks	***			
Harvest	***			
Rootstock × Harvest	**			

	**Seed width (mm)**			

US-802	7.0 ± 0.15 Ca	7.4 ± 0.29 Ba	7.5 ± 0.18 ABa	7.8 ± 0.28 Aa
US-897	5.2 ± 0.15 Cb	5.7 ± 0.08 Abb	5.8 ± 0.19 Ac	5.4 ± 0.16 BCc
US-942	5.9 ± 0.33 ABc	5.8 ± 0.11 Bb	6.2 ± 0.06 ABb	6.3 ± 0.12 Ab
CV (%)	3.13			
Rootstocks	***			
Harvest	***			
Rootstock × Harvest	***			

	**Seed weight (mg)**			

US-802	193.0 ± 4.35 Ca	206.7 ± 5.85 Ba	215.1 ± 5.25 Aa	173.3 ± 4.05 Da
US-897	62.9 ± 3.35 Cc	107.3 ± 2.78 Ac	105.7 ± 4.71 Ac	94.1 ± 4.27 Bc
US-942	99.5 ± 4.62 Cb	136.5 ± 3.64 ABb	137.1 ± 2.53 Ab	131.2 ± 4.55 Bb
CV (%)	3.08			
Rootstocks	***			
Harvest	***			
Rootstock × Harvest	***			

*Means followed by the same capital and lowercase letter in the row and column, respectively, do not significantly differ according to the Tukey’s test (P ≤ 0.05).*

Significant level: **P ≤ 0.01; ***P ≤ 0.001; ns, non-significant.

Seed size, including length and width, was also significantly (*P* ≤ 0.05) variable during fruit maturation. In July, seeds were small for all evaluated rootstocks (Table 3). At that time, a considerable number of seeds had their outer coat (testa) still under the growing stage, thus being more sensitive to damage during storage (Carvalho and Silva, 2013). The seed coat is an important component of the seed, preventing dehydration and premature germination, as it offers resistance to the root apex emergence in the area of the micropyle (Agustí and Primo-Millo, 2020) and also allows seeds to last longer under appropriate storage conditions. The testa appeared totally formed in August for all three varieties, indicating increased seed viability with fruit maturation. Seed weight also fluctuated across the evaluated period reaching the highest weights in August and September for all tested rootstocks. Seed quality parameters, such as size and weight, play a major role in germination and seedling establishment, and are positively correlated with seed vigor, as variability in seed size may contribute to the variance in seed germination performance (Guo et al., 2015).

### Fruit Color Development

**FIGURE 2:**
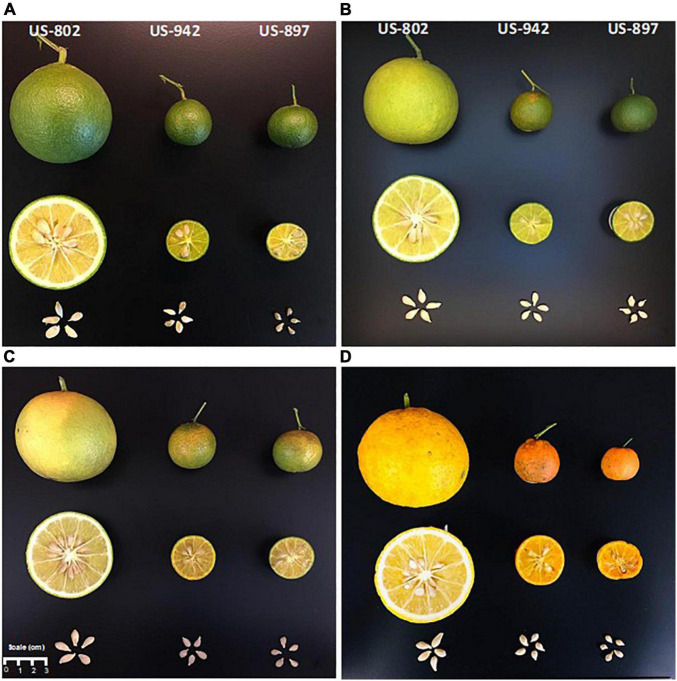
Flavedo color development of citrus rootstock fruits and seed comparison during different harvest periods in mid-July **(A)**, mid-August **(B)**, mid-September **(C)** 2020, and mid-December **(D)** 2019.

**FIGURE 3:**
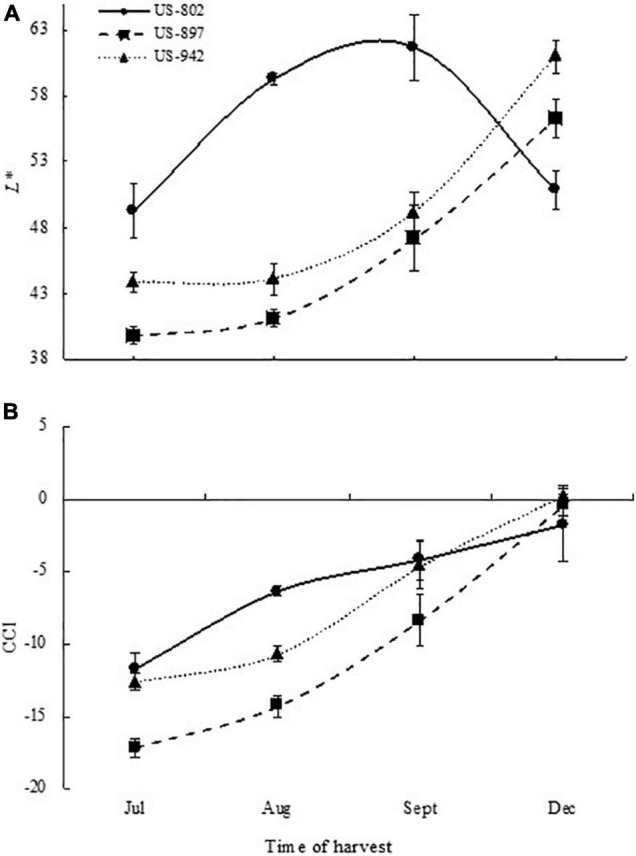
Flavedo color development of citrus rootstock fruits during different harvest periods: Lightness (L*) **(A)** and citrus color index (CCI) **(B)**.

Color is one of the most important parameters to determine maturation of citrus fruit. However, color development in citrus rootstock fruit peel has not gained enough attention, since fruit is not marketable. In our study, we monitored the color change of the peel with the aim of determining if this parameter can be a good non-destructive indicator of seed maturity and hence, viability. Color change in flavedo was monitored during the entire fruit maturation period in all three varieties (Figure 2). The color development was quantitatively described based on lightness (L*) and CCI attributes. The loss of the dark green color and the development of orange color was evident throughout the maturation period (Figures 2, 3). An increase in L* was observed in the flavedo as the fruit collection advanced through the season (Figure 3A). This increase was likely related to the chlorophyll degradation as described by Zhou et al. (2010). Fruit from US-802 had the highest L* value, indicating both a lighter color intensity and earlier peel maturation as compared to fruits from US-897 and US-942. Color break in US-802 and US-942 was observed in August (Figure 2B), a month prior to US-897. By December, the L* decreased only in the US-802 fruits; this might be attributed to the fact that, fruits started to lose water leading to a pressure loss over the peel, which may have affected flavedo lightness. In all varieties, CCI increased during the season. In the case of US-897, color change was delayed as compared to the other two rootstocks and was not evident until September (Figure 2C).

### Seed Germination and Seedling Emergence

In seed lots with high viability, the ability to produce usable seedlings under less-than-optimal germination conditions is related to seed vigor. Seed lots can be classified as low or high vigor, depending on the degree of germination and emergence performance, particularly under unfavorable conditions (Guo et al., 2015). High vigor seeds are proxy of crop establishment and sustainable productivity (Araújo et al., 2016). Thus, seed vigor testing is an essential tool used to evaluate commercial seed lots. The most common vigor tests are based on germination behavior (Hampton and TeKrony, 1995). These include normal germination percentage after a stress imposition, germination speed (time to radicle protrusion), and early seedling growth following germination. Germination speed has been used as an indicator of seed vigor, especially in seed priming experiments (Geneve, 2005), and it is an important measurement used to model seed germination (Bradford, 1990).

**FIGURE 4:**
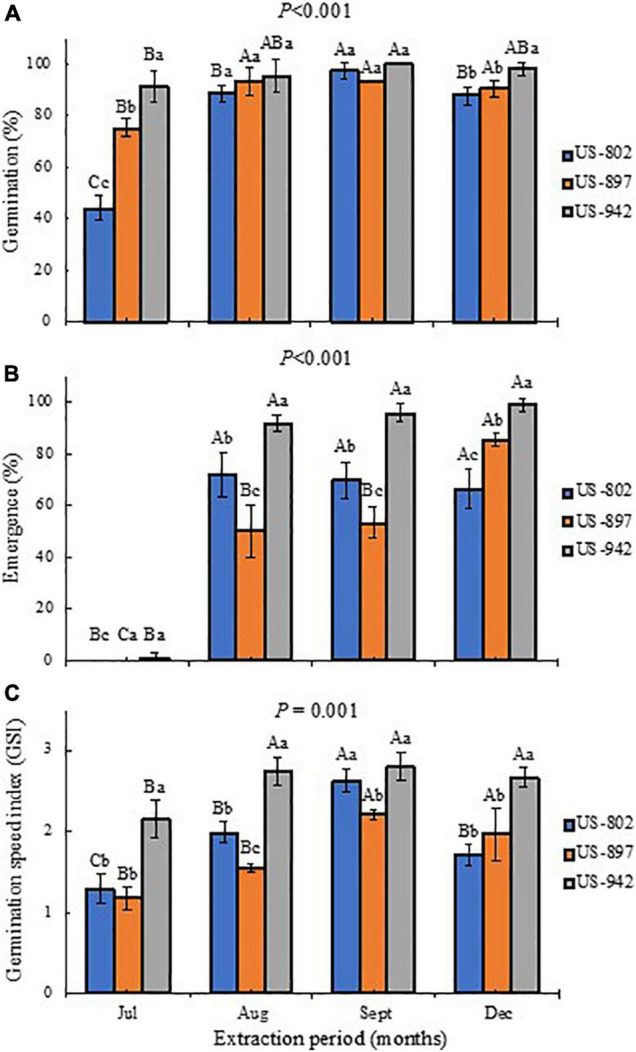
Percentage of germination **(A)**, emergence **(B)**, and germination speed index **(C)** of three citrus rootstocks during different period of seed extraction at the Southwest Florida Research and Education Center, Immokalee, FL, United States. Bars followed by the same lowercase and capital letter do not significantly differ in regard to rootstock and seed extraction period, respectively, according to the Tukey’s test (*P* ≤ 0.05).

The stage of fruit maturation significantly affected the germination performance of the citrus rootstock seeds, irrespective of the variety (Figure 4). There was a significant interaction (*P* ≤ 0.001) between rootstock and time of fruit harvest for almost all physiological characteristics evaluated. Seeds extracted in July had the lowest percentage of germination for all three rootstocks, particularly for the US-802, with less than 50% of the seeds able to germinate (Figure 4A). However, an increase in seed germination was observed when fruit harvest progressed (Figure 4A). Rootstock fruits harvested in August and September had seeds with higher germination potential, as more than 90% of the seeds generated seedlings (Figure 4A), which can be related to the seed quality parameters (size and weight), as larger seeds usually result in improved stand establishment and faster germination (Guo et al., 2015). This rate of germination was similar or even higher than that from the seeds obtained from fully mature fruits, harvested in December.

**FIGURE 5:**
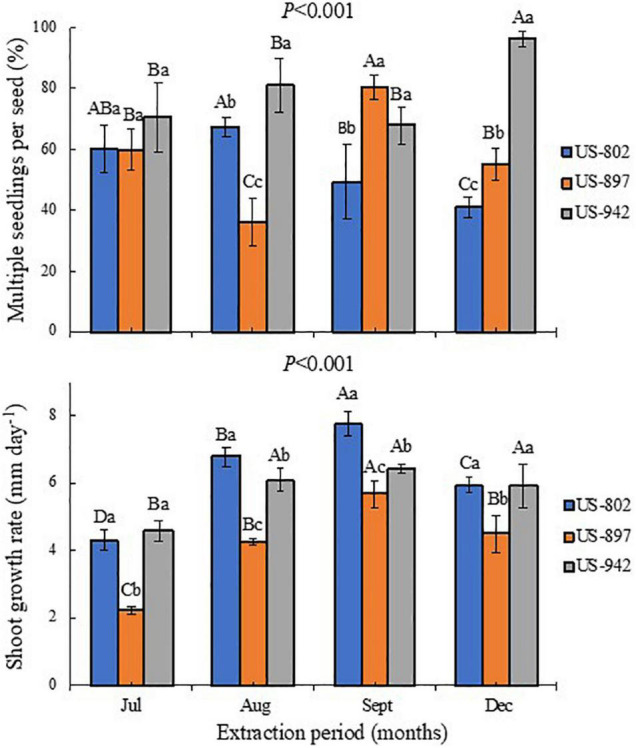
Percentage of multiple seedlings per seed and shoot growth rate of three citrus rootstocks during different period of seed extraction at the Southwest Florida Research and Education Center, Immokalee, FL, United States. Bars followed by the same lowercase and capital letter do not significantly differ in regard to rootstock and seed extraction period, respectively, according to the Tukey’s test (*P* ≤ 0.05).

This germination trend was also observed for seedling emergence, under nursery standard conditions (Figure 4B). In July, the rootstock seedlings did not emerge, indicating that the seeds were not physiologically ready yet to be extracted from the fruits. Later, the seeds performed better, showing good seedling emergence progress, mainly for the US-942 rootstock. In general, seeds from fruits harvested in September showed the highest vigor according to the GSI, and root and SGRs (Figures 4B, 5B and Table 4). All these parameters are important for rootstock seedlings production, as the time-consuming period required in this process is highly dependent on the seed vigor (Bowman et al., 2016a; Vashisth et al., 2020). Based on this, seeds all rootstocks were ready in August, showing US-942 more % of emergence than US-802 and US-897 (Figure 4B), resulting in faster seed germination (Figure 4C); In contrast, US-802 had the faster SGR (Figure 5B). Seeds from US-942 took about 24 days to reach 50% of the total seedling emergence (Table 4). In contrast, seed emergence for the US-897 and US-802 took 37 and 43 days, respectively (Table 4).

**TABLE 4:** Seedling root growth rate and days to reach 50% of the total emergence of three citrus rootstocks during different period of seed extraction at the Southwest Florida Research and Education Center, Immokalee, FL, United States (mean value ± standard deviation).

Source of variance	Root growth rate (mm day^–1^)	T50 (days)^1^
**Rootstock**		
US-802	3.87 ± 0.51 B^2^	43 ± 4.96 A
US-897	3.04 ± 0.54 C	37 ± 5.37 B
US-942	4.61 ± 0.47 A	24 ± 6.43 C
**Harvest**		
July	3.87 ± 0.60 b	38 ± 0.01 a
August	3.74 ± 0.77 b	31 ± 10.57 a
September	4.42 ± 0.67 a	31 ± 8.77 a
December	3.32 ± 0.79 c	39 ± 7.68 a
CV (%)	8.32	24.15
Rootstock	***	***
Harvest	***	***
Rootstock × harvest	ns	ns

*^1^T50, days to reach 50% of the total emergence.*

*^2^Means followed by the same letter in the column do not significantly differ according to the Tukey’s test (P ≤ 0.05).*

Significant level: ***P ≤ 0.001; ns, non-significant.

The seeds of the US-942 rootstock were also more efficient in generating multiple seedlings per seed (Figure 5A), an important aspect to be considered during rootstock production (Bisi et al., 2020). This effect increased as the fruit matured, as compared with the other two rootstocks. In addition to the zygotic embryo, the citrus seeds have nucellar embryos which are developed from somatic nucellus cells (Frost et al., 1968). Polyembryonic seeds usually contain between 2 and 10 nucellar embryos, though, sometimes, they may contain more embryos (Agustí and Primo-Millo, 2020). This characteristic allows to propagate uniform and clonal (true-to-type) rootstock seedlings, as nucellar seedlings are genetically identical to the maternal parent (Kepiro and Roose, 2010). The seed capacity to produce one or more embryos depends on its physiological quality, which is related to the fruit maturation, and nutritional and healthiness conditions of the trees and seeds (Carvalho and Silva, 2013).

### Correlation Analysis

**FIGURE 6:**
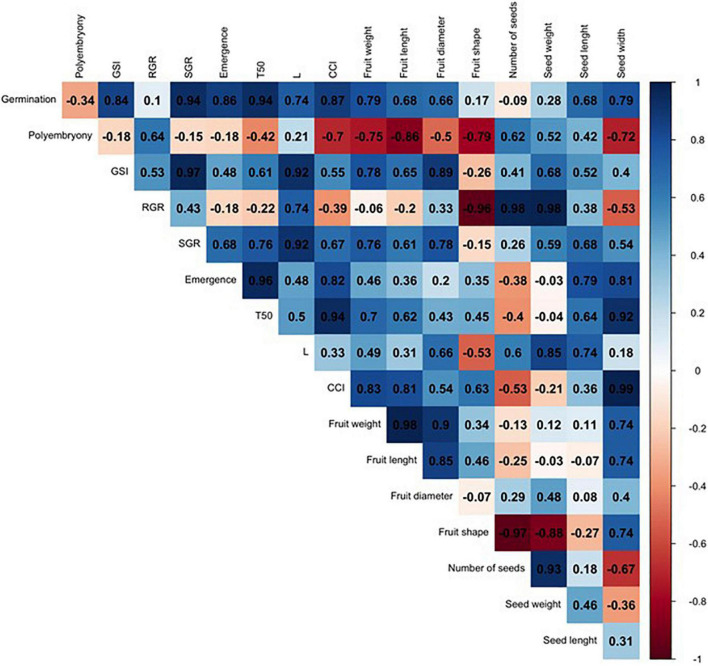
Correlation analysis among the variables studied for US-802 rootstock. The numbers display the Pearson’s correlation coefficient (r). Blue cells indicate positive correlations among the variables (1) and the red cells indicate negative correlations (–1). The color density and numbers reflect the scale of correlation. Variables: germination; polyembryony; GSI: germination speed index; RGR: root growth rate; SGR: shoot growth rate; emergence; T50: days to reach 50% of the total emergence; *L*: lightness value; CCI: citrus color index; fruit weight; fruit length; fruit diameter; fruit shape; number of seeds per fruit, seed weight; seed length; and seed width.

**FIGURE 7:**
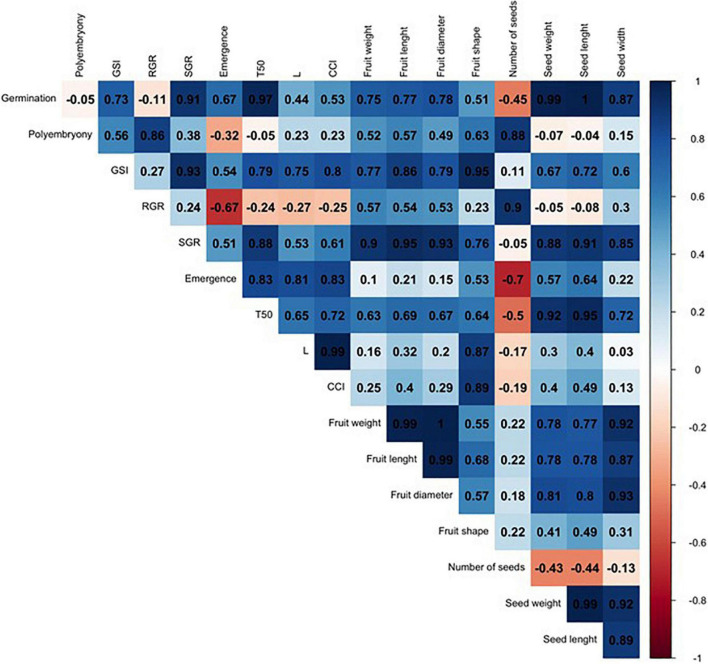
Correlation analysis among the variables studied for US-897 rootstock. The numbers display the Pearson’s correlation coefficient (r). Blue cells indicate positive correlations among the variables (1) and the red cells indicate negative correlations (–1). The color density and numbers reflect the scale of correlation. Variables: germination; polyembryony; GSI: germination speed index; RGR: root growth rate; SGR: shoot growth rate; emergence; T50: days to reach 50% of the total emergence; *L*: lightness value; CCI: citrus color index; fruit weight; fruit length; fruit diameter; fruit shape; number of seeds per fruit, seed weight; seed length; and seed width.

**FIGURE 8:**
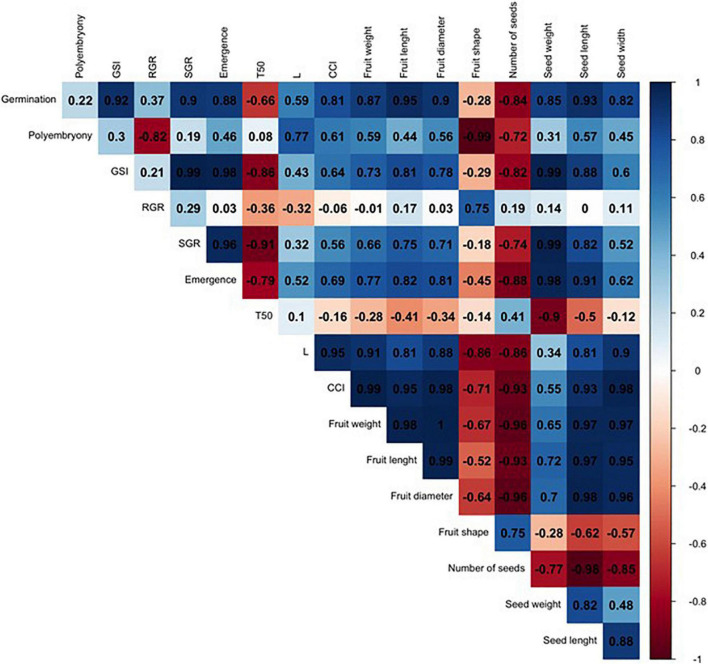
Correlation analysis among the variables studied for US-942 rootstock. The numbers display the Pearson’s correlation coefficient (r). Blue cells indicate positive correlations among the variables (1) and the red cells indicate negative correlations (–1). The color density and numbers reflect the scale of correlation. Variables: germination; polyembryony; GSI: germination speed index; RGR: root growth rate; SGR: shoot growth rate; emergence; T50: days to reach 50% of the total emergence; *L*: lightness value; CCI: citrus color index; fruit weight; fruit length; fruit diameter; fruit shape; number of seeds per fruit, seed weight; seed length; and seed width.

In order to compare such divergent rootstocks, a correlation matrix was built for each single citrus rootstock (US-802, US-897, and US-942) based on Pearson’s correlation coefficients (r) and the studied traits (Figures 6–8 and Supplementary Figure 1). Significant positive correlations (*P* ≤ 0.05) were found (Figure 6 and Supplementary Figure 1) between RGR and seed weight (*r* = 0.98), and number of seeds (*r* = 0.98) for the US-802, but strongly negative with fruit shape (*r* = −0.96). However, no more obvious correlations were observed between all other traits for this citrus rootstock. The correlation analysis for US-897 revealed a strong positive correlation between germination and seed weight (*r* = 0.99, *P* ≤ 0.05), and seed length (*r* = 1.00; *P* ≤ 0.01) (Figure 7), as well as between days to reach 50% of the total emergence (T50) and seed length (*r* = 0.95; *P* ≤ 0.05). No significant negative correlations (*P* ≤ 0.05) were found between the evaluated traits for the US-897. Finally, for US-942 numerous positive and negative correlations were observed among its dimensions (Figure 8). Seed weight was positively (*P* ≤ 0.05) correlated with GSI (*r* = 0.99), SGR (*r* = 0.99), and emergence (*r* = 0.98). Similarly, the CCI was highly and positively correlated with fruit weight (*r* = 0.99; *P* ≤ 0.01), fruit diameter (*r* = 0.98; *P* ≤ 0.05), fruit length (*r* = 0.95; *P* ≤ 0.05), and seed width (*r* = 0.98; *P* ≤ 0.05). Furthermore, the US-942 fruit weight and size (length and diameter) were positively (*P* ≤ 0.05) correlated with seed length (*r* ≥ 0.97) and width (*r* ≥ 0.95) but negatively associated with the number of seeds (*r* ≤ −0.93). Negative correlation was also found for fruit shape and polyembryony (*r* = −0.99; *P* ≤ 0.05).

In general, seed weight was highly correlated with the germination and emergence dimensions for all three evaluated citrus rootstocks. Previous studies have showed strong relationship between seed weight and seedling growth measurements, including germination and emergence, among different species (Hanley et al., 2007; Huang et al., 2017; Poletto et al., 2018). The control of seed mass and size are regulated by genetic and epigenetic pathways, but it has been little investigated in woody perennial plants (Poletto et al., 2018). Current evidence has shown that the homolog of *Auxin Response Factor 19* (*JcARF19*) significantly increases seed size and mass in *Jatropha curcas* plants, confirming the importance of the auxin pathway in controlling these seed attributes in woody perennial plants (Sun et al., 2017). Hanley et al. (2007) reinforce that larger seeds are usually assumed to be more advantageous for seedling establishment than smaller-seeded species, as they are less dependent on soil nutrient availability at the initial growth stage. Indeed, this relationship appears to be triggered by the seed vigor, an important index of seed quality, as larger and heavier seed usually generates more vigorous seedlings (Poletto et al., 2018; Wen et al., 2018). Moreover, this index measures the potential for a rapid and uniform seedling emergence and may reduce seed production risks associated with poor stand establishment (Marcos-Filho, 2015; Wen et al., 2018), resulting in a better management decision.

US-942 seed weight has shown strong relationships with most germination and emergence assessments (Figure 8) evidencing its vigorous potential as have been reported previously (Bowman and Joubert, 2020). Furthermore, this rootstock provided superior field performance to various other high-demanded citrus rootstocks under Florida soil-climate condition and HLB-pressure (Bowman et al., 2016a,b).

### Assessing Abscission Capacity

**FIGURE 9:**
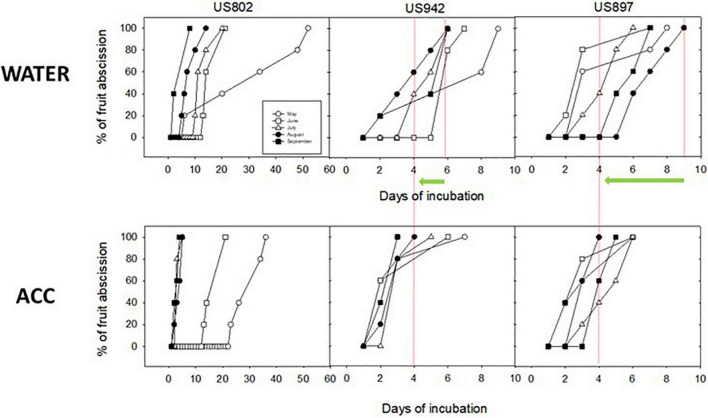
Abscission dynamics of fruit from US-802, US-897, and US-942 harvested on May, June, July, August, and September, and incubated *in vitro* in the absence (lower panels, water control) or presence (upper panels) of the ethylene precursor ACC (1-aminocyclopropane-l-carboxylic acid). The 3 panels are aligned to allow comparisons of abscission rate. Green arrows and red lines highlight the advancement in fruit abscission rate for August.

Color data, when taken together and compared with seed emergence and germination data suggest that color change in the peel is not the best indicator of seed maturity for these rootstock varieties, because on one hand, fruit from US-802 changed color 1 month earlier than fruit from US-897, but seed germination was not different by August, and on the other hand, fruit from US-942 and US-802 changed color at the same time, but GSI in US-942 were significantly higher than in US-802. With maturation, fruit enters senescence and ultimately abscission, two processes that are genetically determined and hormonally regulated. Citrus fruits, although non-climacteric, can respond to ethylene, and this response is highly coordinated and may involve abscission (Alferez and Zacarias, 1999; Alférez et al., 2006; Alferez et al., 2021b); in this sense, increased capacity for abscission could indicate when the seed is ready to germinate, as fruit abscission is a mechanism for seed dispersal in nature. To further confirm this idea, we studied *in vitro* the fruit abscission response during maturation in the three rootstocks. We compared the abscission response of fruit from the three rootstocks to the ethylene precursor ACC during maturation in time course experiments monitoring abscission rate. We found that all three rootstocks started to respond to ACC in advancing abscission as early as July, but response was maximized in August; Fruit from US-942 incubated with water (Figure 9 upper central panel) reached 100% abscission by day 6 of incubation, whereas after ACC treatment this time was reduced to 4 days; in the case of US-897, the abscission response after ACC treatment was advanced from 9 to 4 days in fruit harvested in August, whereas in fruit from US-802 abscission was advanced from 10 to 7 days, showing a milder response to the ethylene precursor This set of data further supports the notion that seeds from all three varieties may be ready to be harvested as early as August (Figure 9).

## Conclusion

The physiological response on rootstock seed germination is affected by the maturation of the fruits at harvest. For Florida conditions, fruits from all three varieties can be harvested as early as August without losing any germination potential based on seed vigor parameters studied here and on our study of abscission dynamics. Therefore, fruits from these rootstock varieties can be harvested for seed extraction before the peak of the hurricane season, avoiding fruit drop and eliminating the risk of not having enough seeds for nursery supply. Together, this constitutes a new managerial tool for nurseries in Florida to control and adjust operations.

## Data Availability Statement

The original contributions presented in the study are included in the article/supplementary material, further inquiries can be directed to the corresponding author/s.

## Author Contributions

FA conceived the project and got the funding. DC, DB, and TG performed the research. DC, FA, and RL analyzed the data. DC and FA wrote the manuscript. All authors accepted the final manuscript.

## Conflict of Interest

The authors declare that the research was conducted in the absence of any commercial or financial relationships that could be construed as a potential conflict of interest.

## Publisher’s Note

All claims expressed in this article are solely those of the authors and do not necessarily represent those of their affiliated organizations, or those of the publisher, the editors and the reviewers. Any product that may be evaluated in this article, or claim that may be made by its manufacturer, is not guaranteed or endorsed by the publisher.

## Supplementary Material

Supplementary Figure 1 Scatterplot and correlation matrix of the studied variables for US-802, US-897, and US-942 rootstocks. The matrix contains of pairwise scatterplots for the following variables: GERM: germination; POLY: polyembryony; GSI: germination speed index; RGR: root growth rate; SGR: shoot growth rate; EMER: emergence; T50: days to reach 50% of the total emergence; L: lightness value; CCI: citrus color index; FW: fruit weight; FL: fruit length; FD: fruit diameter; FS: fruit shape; NS: number of seeds per fruit; SWe: seed weight; SL: seed length; and SWi: seed width. Significant level: ^∗^*P* ≤ 0.05; ^∗∗^*P* ≤ 0.01; ^∗∗∗^*P* ≤ 0.001.
