# Supplementary figures and images for: Determining Seed Viability During Fruit Maturation to Improve Seed Production and Availability of New Citrus Rootstocks
Source: Front Plant Sci. 2021 Nov 19;12:777078. doi: 10.3389/fpls.2021.777078 (PMC8641648; doi:10.3389/fpls.2021.777078)

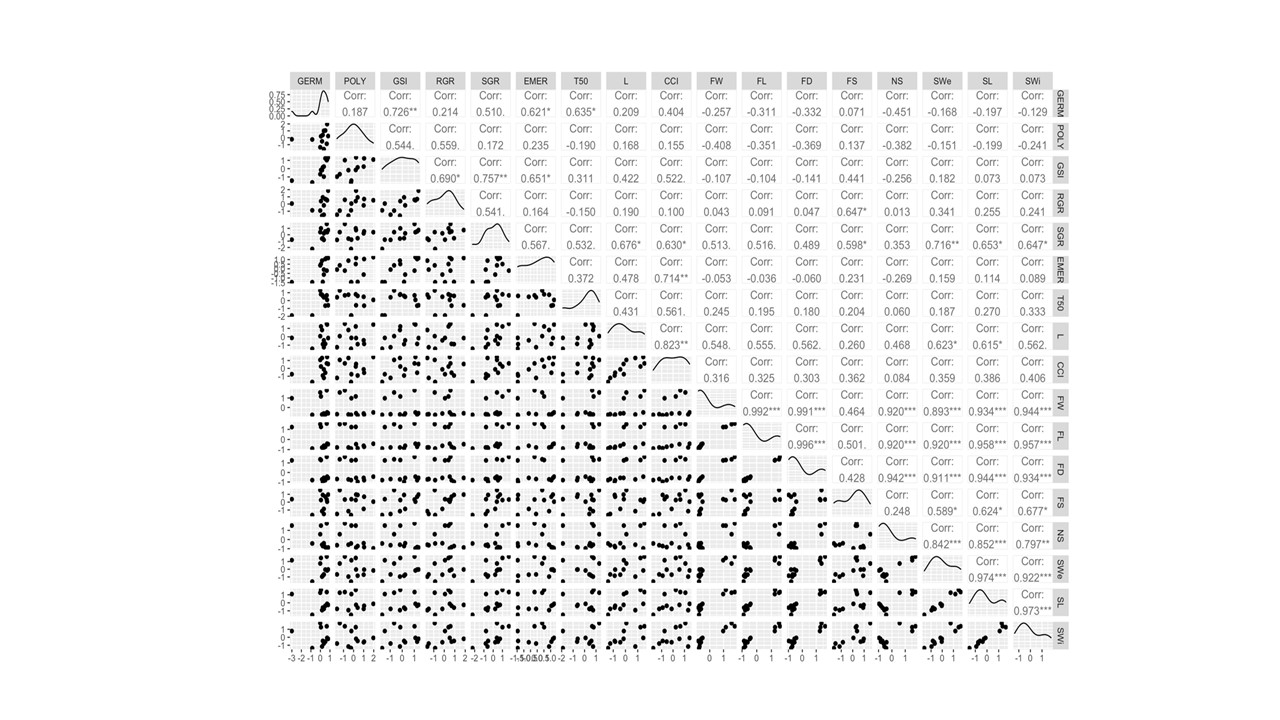

Supplement: Supplementary Figure 1 — Scatterplot and correlation matrix of the studied variables for US-802, US-897, and US-942 rootstocks. The matrix contains of pairwise scatterplots for the following variables: GERM: germination; POLY: polyembryony; GSI: germination speed index; RGR: root growth rate; SGR: shoot growth rate; EMER: emergence; T50: days to reach 50% of the total emergence; L: lightness value; CCI: citrus color index; FW: fruit weight; FL: fruit length; FD: fruit diameter; FS: fruit shape; NS: number of seeds per fruit; SWe: seed weight; SL: seed length; and SWi: seed width. Significant level: ∗P ≤ 0.05; ∗∗P ≤ 0.01; ∗∗∗P ≤ 0.001. [file Image_1.JPEG]
